# Global household air pollution database: Kitchen concentrations and personal exposures of particulate matter and carbon monoxide

**DOI:** 10.1016/j.dib.2018.10.120

**Published:** 2018-10-27

**Authors:** Matthew Shupler, Kalpana Balakrishnan, Santu Ghosh, Gurusamy Thangavel, Sasha Stroud-Drinkwater, Heather Adair-Rohani, Jessica Lewis, Sumi Mehta, Michael Brauer

**Affiliations:** a School of Population and Public Health, University of British Columbia, Vancouver, British Columbia, Canada; b Department of Environmental Health Engineering, Faculty of Public Health, Sri Ramachandra Medical College and Research Institute (Deemed to be University), Chennai, India; c Department of Biostatistics, St Johns Medical College, Bangalore, Karnataka, India; d World Health Organization, Geneva, Switzerland; e Vital Strategies, New York, New York, United States

## Abstract

The Global Household Air Pollution (HAP) Measurements database, commissioned by the World Health Organization, provides an organized summary of data reported in the literature describing HAP microenvironments, methods and measurements. As of June 2018, the database contains measurements from 43 countries obtained from 196 studies published through 2016. The database includes information useful for understanding the range of household and personal air pollution measurements that have been collected in a country, as well as characteristics of the cooking environment, including primary cooking fuel type, stove type, heating fuel type and kitchen location. Quantitative particulate matter (PM) of various size fractions and/or carbon monoxide (CO) exposure measurements included in the database can be aggregated and analyzed to generate summary statistics (e.g. average sub-national, national, regional and global HAP exposures) to assess temporal and spatial relationships. The quantitative PM exposure measurements in the database have been used in global predictive modeling of HAP-PM_2.5_ exposures (“Global Estimation of Exposure to Fine Particulate Matter (PM2.5) from Household Air Pollution” (Shupler et al., 2018) [1])

**Specifications table**

**Table:** 

Subject area	Environmental health, Exposure science, Air pollution epidemiology
More specific subject area	Household air pollution, Cooking and heating with clean and polluting fuels and technologies, Household solid fuel use, Indoor air quality / pollution
Type of data	.xls spreadsheet
How data was acquired	Systematic search of PubMed and Science Direct literature and manual data extraction from identified studies from 1981 to 2016
Data format	Analyzed
Experimental factors	Please include a brief description of preparation (required)
	A literature search using PubMed (National Library of Medicine and National Institute of Health) and the Science Direct (Elsevier) search engines was used to identify suitable publications. Articles were scanned for eligibility based on existence of quantitative HAP measurements. Relevant data was extracted and entered into the database.
Experimental features	Please include a brief description of the data features (required)
	Qualitative information includes cooking environment descriptions (e.g. primary cooking fuel type, stove type, heating fuel type and kitchen location), monitoring period (e.g. 24, 48 h), monitoring method (e.g. gravimetric, light scattering). Quantitative information includes cooking area concentrations and personal exposure measurements for PM and CO.
Data source location	Data available from 43 countries across Asia, Africa, Central/South America, and, to a lesser extent, Europe and North America
Data accessibility	Data is with this article and available at the World Health Organization Global HAP Measurements Database: http://www.who.int/airpollution/data/hap-measurements/en/
Related research article	[1] Shupler, M; Godwin, W; Frostad, J; Gustafson, P; Arku, RE; Brauer, M. Global Estimation of Exposure to Fine Particulate Matter (PM2.5) from Household Air Pollution. *Environment International 2018; 120:354-363.*

**Value of the data**

• Serves as the most comprehensive repository of HAP measurement studies that can be useful when seeking to comprehensively review existing HAP exposure literature [2], [3].
• Allows for merging of quantitative data across studies to summarize HAP exposures across geographical regions and/or populations of interest (men, women, children).
• Informs what type of technology assessments are commonly carried out in quantitative HAP exposure studies.
• Describes the type of qualitative data commonly collected about the cooking environment in HAP studies.
• Identifies countries and regions where HAP studies have historically been conducted, which can in turn be used to identify areas where little to no HAP studies currently exist.

## 1 Data

The data file is a single excel spreadsheet with over 1000 rows of quantitative HAP measurements that have been compiled from published articles. Citations corresponding to each measurement are provided in each row. For each peer-reviewed article with measurements contained in the database, the first row of entry is labeled as “General Study Descriptives” and provides qualitative information on the cooking environment/participants of the study described by the article. Subsequent rows for each article each refer to a unique quantitative measurement obtained from the article. Quantitative measurements are separated in different columns by pollutant: “PM Measurement Results” and “CO Measurement Results”. If multiple measurements were reported in the same study, the study will have multiple rows in the database. The number of rows corresponding to a study are labeled in the database.

• **General Study Information** (Columns A through AO)
  a. Study setting (rural/urban, season)
  b. Study design
  c. Primary cooking fuel, heating fuel, stove type
  d. Kitchen location, kitchen type, family size
  e. Ventilation
  f. Number of participants, age & gender of participants
• **Detailed Particulate Matter (PM) Measurements** (Columns AP through BV)
  a. PM size fraction
  b. Number and type of measurement (e.g. kitchen, personal, ambient)
  c. Measurement method (e.g. gravimetric, light scattering)
  d. Averaging period and unit of measurement
• **Detailed Carbon Monoxide (CO) Measurements** (Columns BW through CX)
  a. Number and type of measurement (e.g. kitchen, personal, ambient)
  b. Measurement method
  c. Averaging period and unit of measurement
• **Ratios Related to PM–CO Measurements** (Columns CY to DE)
  a. Ratios of area concentrations (e.g. kitchen to living) and area concentrations to personal exposures
  b. PM/CO ratios and correlation coefficients
• **PM /CO Models** (Columns DF to DN)
  a. Model variables
  b. Significant model determinants
  c. Model R^2^
• **Air Toxics/Biomarker Measurement Results** (Columns DO to EP)
  a. Number and type of measurement
  b. Measurement method
  c. Location of sampler
  d. Averaging period and unit of measurement
• **Health Assessments included with Exposure Measurements** (Columns EQ to EV)
  a. Health endpoint assessed
  b. Epidemiological measure and strength of association (where applicable)
• **Measurement protocols and quality control information** (Columns EW to EX)
• **Miscellaneous / Rarely Reported Information** (Columns EY to FJ)
  a. Fuel moisture content
  b. Cooking length measurement type (e.g. time-activity diary, stove use monitor)
  c. Improve cookstove cost/likeability among participants (where applicable)
  d. Light scattering/gravimetric correction factor and correlation

## 2 Experimental design, materials, and methods

Peer-reviewed articles included in the data base were identified via a literature review using PubMed (National Library of Medicine and National Institute of Health) and Science Direct (Elsevier) search engines. Key terms included in the literature search are available elsewhere [4]. Identified articles were scanned for quantitative HAP measurements and data was entered manually.
